# Alcoholism, Tuberculosis and Venereal Diseases as Factors in Stirpiculture

**Published:** 1913-08

**Authors:** 


### Alcoholism, Tuberculosis and Venereal Diseases as Factors in Stirpiculture

We have been impressed with the frequency with which alcoholism, tuberculosis and syphilis are emphasized in the French literature as preponderating influences in determining the ultimate health and viability of the offspring. Certain observations have led us to question whether the first is so often as it is usually believed, operative in establishing a hereditary tendency to drink. Several of the strictest abstainers that we have known have, in a sense, inherited this negative tendency from parents with the opposite, positive tendency, on exactly the same principle that the children of profligate or dishonest parents, with the constant warning before them and the reiterated precepts of the other parent, become models of thrift and scrupulous adherents of truth and respecters of the rights of others. Nor do we believe that moderate drinkers often degenerate into drunkards or that occasional excessive drinkers establish characteristic lesions in their system, much less well defined hereditary physical taints. More important are the general effects of malnutrition and exposure producing no special lesion in the offspring but a generally enfeebled constitution; and the almost inevitable financial drawbacks, amounting to inadequate care of children and virtually constituting a partial orphanage. Yet we recall a really admirable man, who conducted a business with great success and who was scarcely out of the influence of alcohol for twenty years, except early in the morning, when he promptly brought himself into his habitual if not strictly normal condition. This man and his wife held the highest ethical views of propagation, and the latter was about as constantly in the stage of gestation or lactation as her husband was under the moderate influence of alcohol. The net result was two normal children, one feeble one and one markedly anaemic and below par intellectually; as well as a long row of little graves and many innocent abortions.

The extent to which tuberculosis is hereditary is still a debatable question. The disease is so common that practically no one who is a member of a large family and who knows anything about its history can fail to give what might, at least, be construed as a collateral heredity. Frank congenital tuberculosis is rare and the degree to which subsequent development of the disease should be ascribed to ante-natal but latent infection, the inheritance of a definite diathesis, or merely to subsequent familial or extra-familial infection, is still in doubt. Viewed from the practical standpoint, tuberculosis of a clinically recognizable and dangerous degree, is rather a disease of lowered resistance than analogous to the majority of germ-diseases (we say *germ* because both bacteria, protozoa and other micro-organisms are concerned) in which infection, granted the presence of the infecting organisms, depends very little on the general health and strength of the individual. While instances may be multiplied to show that a weak child, born of tuberculous parents, may by proper regimen in a different environment not only escape the disease but become vigorous, the offspring of the tuberculous is, as a rule, feeble and especially exposed to infection.

The constant struggle against alcoholism and tuberculosis is unquestionably mitigating these evils. Every case of tuberculosis prevented or cured makes for healthier offspring, for better fed, better clothed and more carefully watched children, and it actually lessens the danger of infection during periods of temporary depression of resistance such as are inevitable in any individual. We venture the prediction that tuberculosis will decrease in geometric progression and that, if present or more effective measures of control are maintained for a generation, it will become a relatively rare instead of one of the commonest diseases. Meantime, various philanthropic agencies are at work to preserve children from what were formerly almost inevitable results of alcoholism, tuberculosis and other unfavorable factors of parental origin.

The problems of syphilis are inevitably complicated with moral considerations. There still survive many who regard the prophylaxis of venereal disease as immoral. If syphilis were entirely a disease of venereal origin and if it involved only the trespassers of social law, there would be some excuse for this attitude. But, in the face of well known facts, we may boldly advocate taking every measure to prevent syphilis or any other venereal disease, with no more attention to what the recipient might be disposed to deserve than in the case of typhoid fever, which ultimately involves a violation of rules of decency along other lines, or of traumatisms to which individual carelessness contribute. As to the methods to be followed, we have nothing to say here, except the following general heresies: Too detailed warnings to our youth as to the punishment of immorality may lead to attempts, not altogether successful, to avoid not the sin but its untoward results. There is very little hope of eradicating an institution that has existed for the last five thousand years or longer, by prohibitive legislation. The prostitute is only one factor in maintaining this institution, and there is neither justice nor common sense in persecuting her as "disorderly" simply on account of her business. Without advocating seggregation in the formal sense, we believe that flies, filth and foci of immorality are much less dangerous in visible masses than when scattered. There is no use sticking our heads into the sand and imagining that we have annihilated prostitution.

